# Molecular Sensing by Nanoporous Crystalline Polymers

**DOI:** 10.3390/s91209816

**Published:** 2009-12-03

**Authors:** Pierluigi Pilla, Andrea Cusano, Antonello Cutolo, Michele Giordano, Giuseppe Mensitieri, Paola Rizzo, Luigi Sanguigno, Vincenzo Venditto, Gaetano Guerra

**Affiliations:** 1 Optoelectronic Division, Engineering Department, University of Sannio, Benevento 82100, Italy; E-Mails: pillapie@unisannio.it (P.P.); a.cusano@unisannio.it (A.C.); cutolo@unisannio.it (A.C.); 2 Institute of Composite Materials Technology National, Research Council (ITMC-CNR), Portici, Napoli 80055, Italy; E-Mail: g.michele@unina.it; 3 Department of Materials and Production Engineering and INSTM Research Unit, University of Naples Federico II, P.le Tecchio 80, 80125 Naples, Italy; E-Mail: mensitie@unina.it; 4 Chemistry Department and INSTM Research Unit, University of Salerno, Fisciano, 84084, Italy; E-Mails: prizzo@unisa.it (P.R.); vvenditto@unisa.it (V.V.); 5 Technological District on Polymeric and Composite Materials Engineering and Structures—IMAST, Piazzale E. Fermi 1, Località Granatello, 80055 Portici (Na), Italy; E-Mail: lusangui@unina.it

**Keywords:** polymer co-crystals, nanoporous crystalline phases, syndiotactic polystyrene, sensing film rigidity, molecular sensors, chiral sensors

## Abstract

Chemical sensors are generally based on the integration of suitable sensitive layers and transducing mechanisms. Although inorganic porous materials can be effective, there is significant interest in the use of polymeric materials because of their easy fabrication process, lower costs and mechanical flexibility. However, porous polymeric absorbents are generally amorphous and hence present poor molecular selectivity and undesired changes of mechanical properties as a consequence of large analyte uptake. In this contribution the structure, properties and some possible applications of sensing polymeric films based on nanoporous crystalline phases, which exhibit all identical nanopores, will be reviewed. The main advantages of crystalline nanoporous polymeric materials with respect to their amorphous counterparts are, besides a higher selectivity, the ability to maintain their physical state as well as geometry, even after large guest uptake (up to 10–15 wt%), and the possibility to control guest diffusivity by controlling the orientation of the host polymeric crystalline phase. The final section of the review also describes the ability of suitable polymeric films to act as chirality sensors, *i.e.*, to sense and memorize the presence of non-racemic volatile organic compounds.

## 1. Introduction

Chemical sensors play an important and growing role in diverse fields, including environmental (ground water and air pollution) monitoring, toxic chemical agent detection and medical diagnosis. In recent years, great efforts have been dedicated to realizing chemical sensors based on the integration of appropriate sensitive layers and suitable high-sensitivity transducing mechanisms. In general, the sensitive element should optimize specific interactions with a target analyte, provide a fast and reversible diffusion of the analyte and small recovery times, and maintain their physical state as well as their geometry over several cycles of use, in order to avoid hysteresis effects, thus ensuring reproducibility.

Many sensing elements are based on porous materials and use the changes in physical properties that occur when the pores are occupied by the analyte species [1-17]. Although inorganic porous materials [1-6] (mainly silicon) [1-3] can be effective, there is significant interest in extending these concepts to polymeric materials because of their comparatively easy fabrication process, cost effectiveness and mechanical flexibility.

Several studies have shown that porous polymeric absorbents characterized by meso-or macroporous amorphous phases can be used as sensing elements in combination with several kinds of transducing mechanisms [7-10]. Sensing amorphous porous polymers generally present poor selectivity and in some cases their selectivity has been increased by molecular imprinting [11-13]. To increase sensitivity and response rate, nanostructured polymers (mainly nanofibers [14,15] and materials based on block copolymers [16]) have also been proposed as molecular sensing elements. It is also worth adding that micropatterned polymeric grating structures have been demonstrated as suitable platform for recognition elements [17].

A different class of nanoporous polymers, exhibiting crystalline (and hence all identical) rather than amorphous nanopores, has been recently proposed as selective molecular sensing materials. In particular, all reported studies refer to the nanoporous crystalline phases of syndiotactic polystyrene (s-PS), a robust commercial stereoregular polymer.

The first part of this review presents basic information on s-PS, mainly describing the structure and properties of its two nanoporous crystalline phases. The following section describes transport properties of vapours and gases into semicrystalline s-PS films as well as the dependence of mechanical properties on guest sorption. Two following sections describe the use of s-PS films, presenting the nanoporous crystalline phases, as sensing elements of gravimetric and fiber-optic sensors, which are suitable for detection of volatile organic pollutants (mainly chlorinated and aromatic being present in industrial wastes like, e.g., benzene, toluene, chloroform, methylene chloride, dichloroethane, tetrachloroethylene and trichloroethylene) as well as of relevant gases (like ethylene and carbon dioxide). It is also shown that films presenting a nanoporous crystalline phase present as an additional advantage the possibility to control the orientation of the nanopores with respect to the film surface and hence to control the diffusivity of the analytes. The final section of the review describes the ability of s-PS films, when prepared by suitable processes, to act as chirality sensors, *i.e.*, to sense and memorize the presence of non-racemic volatile organic compounds.

## 2. Nanoporous Crystalline Phases of s-PS

### 2.1. Polymorphism of s-PS

Syndiotactic polystyrene (s-PS), whose synthesis was reported about two decades ago [18,19] is a stereoregular polymer which is easily crystallizable (typical degrees of crystallinity are in the 30–50% range 0 and presents a very complex polymorphic behavior [19-23].

In particular, s-PS can form two crystalline phases (α [24,25]and β [26,27]) exhibiting trans-planar chains, when crystallized by melt-processing or by thermal treatments on glassy samples. Although their density are substantially different (ρ_α_ ≈ 1.04 g/cm^3^, ρ_β_ ≈1.08 g/cm^3^) both present similar high melting temperatures (∼270 °C). On the other hand, crystallization procedures from solutions, in most cases, lead to the formation of co-crystalline phases of s-PS with low molecular mass guest molecules, where the host polymer chains present the helical s(2/1)2 conformation [28-35].

Guest removal, by thermal procedures in the temperature range 100–140 °C, from these co-crystalline phases generally produces the γ crystalline phase [19,21,36,37], exhibiting the same helical conformation and high packing density (ρ_γ_ ≈ 1.08 g/cm^3^). Guest removal by suitable solvent extraction procedures can lead to the formation of two additional helical crystalline phases (δ [38-42] and ε [43-45]), which present a low packing density (ρ_δ_ ≈ ρ_ε_ ≈ 0.98 g/cm^3^) and have been described as nanoporous.

The term of polymeric nanoporous phase (and sometimes the term “polymeric framework”) is used to indicate crystalline phases, whose density is lower than the density of the corresponding amorphous phase (as for s-PS, ρ_am_ ≈ 1.05 g/cm^3^) and being suitable to absorb guest molecules at low activities (e.g., from diluted solutions).

To complete the basic information on s-PS polymorphism, it is worth adding that two mesomorphic phases presenting trans-planar [46-48] and helical [40,49] chains have also been found and characterized. Both nanoporous δ and ε phases are irreversibly transformed into the dense helical γ phase by annealing procedures in the range 90–120 °C. The ε→γ transition occurs directly while the δ→γ transition occurs with the formation, for intermediate temperatures, of the helical mesomorphic phase. It is also worth adding that also the γ phase is thermally unstable and by treatments above 180 °C is irreversibly transformed in a trans-planar crystalline phase (generally the α phase).

### 2.2. The Nanoporous δ Phase of s-PS

**Figure 1.**
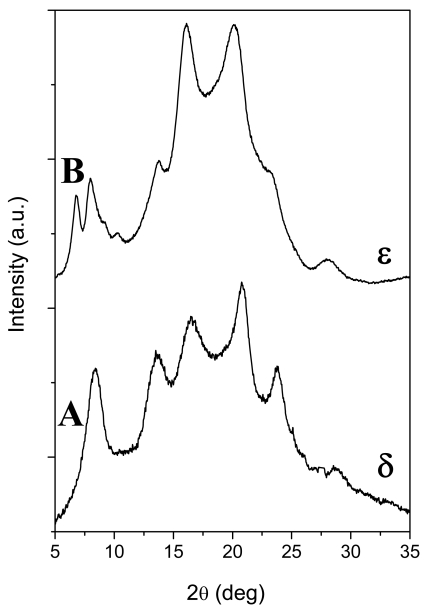
X-ray diffraction patterns (CuKα) of s-PS semicrystalline powder samples presenting the two nanoporous crystalline phases: (A) δ-form; (B) ε form.

The first nanoporous polymeric crystalline phase, the δ phase of s-PS, was discovered and patented in 1994 [42]. It can be easily obtained from most s-PS co-crystalline phases by guest-extraction procedures by solvents which are its temporary volatile guests, like e.g., acetone, acetonitrile [50] or supercritical carbon dioxide [51,52]. The X-ray diffraction pattern of an unoriented sample exhibiting this crystalline phase is shown in Figure 1A and can be easily distinguished by an intense peak corresponding to the Bragg distance *d* = 1.06 nm (2θ_CuKα_ ≈ 8.4°) and by very weak peaks in the 2θ_CuKα_ range 9.5°–12°.

**Figure 2.**
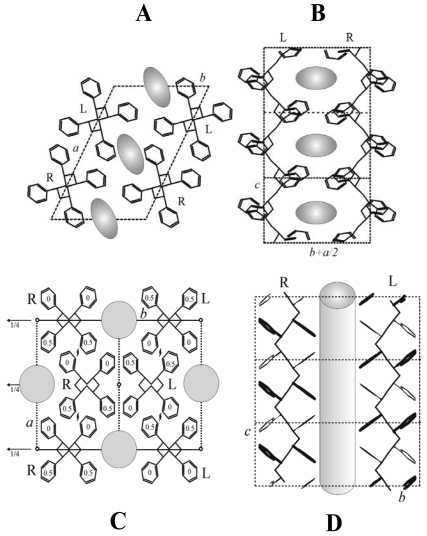
Top and lateral views of the crystalline structures of the two nanoporous crystalline phases of s-PS: for the δ (upper figures) and ε (lower figures) phases, the porosity is distributed as cavities and channels, respectively.

**Figure 3.**
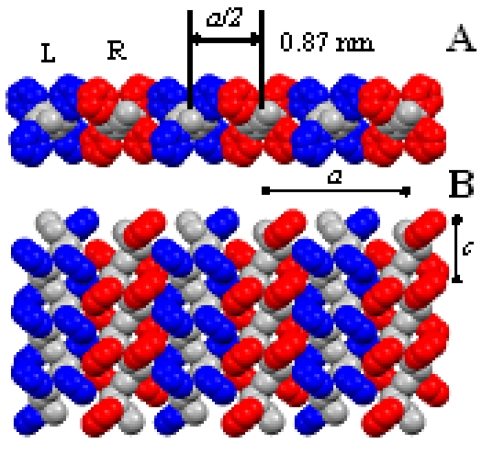
Top (A) and lateral views (B) of the *ac* layer of s(2/1)2 helices of s-PS, *i.e.*, the high density and low-energy structural feature which is common to the δ nanoporous form and to the corresponding co-crystalline forms. The minimum interchain distance (0.87 nm) is achieved by alternating enantiomorphous helices (R and L stand for right-handed and left-handed, respectively).

A few years later, the crystal structure of the δ form has been determined by the analysis of the X-ray fiber diffraction pattern and packing energy calculations. Two chains in the helical s(2/1)2 conformation are packed in the monoclinic unit cell with axes *a* = 1.74 nm, *b* = 1.185 nm, *c* = 0.77 nm, and γ = 117°, with a density of 0.98 g cm^−3^, according to the space group *P*21/*a* (Figure 2A-B) [38]. The structure is similar to the model proposed for some s-PS clathrate co-crystalline structures [28-30]: the *b* axis is shorter and the distance *b* sinγ between *ac* layers of macromolecules (also shown in Figure 3) is shortened to 1.056 nm, as a consequence of the removal of the guest molecules.

The empty space for the δ nanoporous form corresponds to cavities (two per unit cell) centered on the center of symmetry of the crystal structure. The cavity is rather flat, *i.e.*, it presents its maximum dimension (nearly 0.8 nm) nearly perpendicular to the polymer chain axis (essentially along the *a-b* direction) while its minimum dimension (nearly 0.3 nm) essentially along the *c* axis (Figure 2A,B) [39]. In the δ form unit cell, the maximum number of guest molecules per cavity is a well defined integer (generally one or two, depending on guest molecular volume) [28-31]. For most planar guests, the molecular planes are roughly perpendicular to the polymer chain axis.

### 2.3. The Nanoporous ε Phase of s-PS

The nanoporous ε phase of s-PS was discovered only in 2007 and can be obtained by chloroform sorption and desorption in γ form samples [43-45,53]. Its typical X-ray diffraction pattern, for unoriented samples presents, in the low 2θ range, two well defined reflections for Bragg distances *d* = 1.28 nm and 1.08 nm (2θ_CuKα_ ≈ 6.9° and 8.2° in Figure 1B), rather than the single peak at *d* = 1.06 nm typical of the δ phase (Figure 1A) [43-45,53].

The analysis of the reflections of patterns obtained for axially oriented samples indicates the occurrence of an orthorhombic unit cell with axes *a* = 1.61 nm, *b* = 2.18 nm and *c* = 0.79 nm, with four chains of s-PS in the **s**(2/1)2 helical conformation and a density of 0.98 g cm^−3^ (space group *Pbcn*) [45]. The model of packing corresponding to the minimum of the packing energy (Figure 2C-D) is characterized by channel-shaped cavities crossing the unit cells along the *c* axis and delimited, along *b* axis, by two enantiomorphic helical chains.

In these channels, planar guest molecules can be hosted with their molecular planes roughly parallel to the polymer chain axis. Moreover, the presence of channels allows the sorption of guest molecules presenting a molecular axis much longer than the s-PS chain axis periodicity, like e.g., 4-(dimethylamino)cinnamaldehyde, which are not absorbed by the δ nanoporous phase [43,44].

### 2.4. Three Different Uniplanar Orientations of Co-crystalline and Nanoporous Phases of s-PS

s-PS processing in the presence of suitable solvents can lead to the unprecedented formation of co-crystalline films exhibiting three different kinds of uniplanar orientations of the co-crystalline phases [54-60]. The degree and the kind of uniplanar orientation depends on the selected technique (solution crystallization procedures or solvent induced crystallization in amorphous samples or solvent induced re-crystallizations of γ and α unoriented samples [59]) as well as on the chemical nature of the guest.

It has been recently suggested that the structural feature determining these three different kinds of uniplanar orientations is the layer of close-packed alternated enantiomorphous helices [60] (Figure 3) that characterizes the δ phase of s-PS, (Figure 2A) as well as all related co-crystalline phases with low-molecular-mass guest molecules.

In fact, the three observed uniplanar orientations correspond to the three simplest orientations of the high planar-density *ac* layers (Figure 3) with respect to the film plane, which have been named *a_∥_ c_∥_, a_∥_ c*_⊥_ and *a*_⊥_ c*_∥_*, indicating crystalline phase orientations presenting the *a* and *c* axes parallel (*∥*) or perpendicular (⊥) to the film plane [60].

The three uniplanar orientations (without substantial loss of their degree of orientation) are maintained not only for the δ phase, as obtained by guest-removal procedures, but also for the γ phase [19,37,54] as obtained by thermal treatments [54,57,58]. Three different kinds of uniplanar orientations have been recently obtained also for the nanoporous ε phase [61].

The availability of s-PS films with three different kinds of uniplanar orientation not only allows establishing fine structural features of s-PS crystalline and co-crystalline phases (e.g., experimental evaluation of the orientation of transition moment vectors of host and guest vibrational modes, with respect to the host chain axes) [62,63] but can be relevant also for practical purposes. For instance, it allows guest orientation control [64-68] for co-crystalline phases and guest diffusivity (and hence permeability) control [66-69] (see Section 3) for the nanoporous phases.

## 3. Mass Transport and Sorption Thermodynamics of Low Molecular Weight Compounds

### 3.1. Guest Sorption Isotherms in Nanoporous Crystalline s-PS Samples

The sorption behaviour of low-molecular weight compounds in semi-crystalline polymeric materials is usually assumed to occur only in the amorphous domains; in fact, diffusants are not likely to penetrate and dissolve in the crystalline phase, which is usually denser than the amorphous one. As a consequence, in most of the cases, solubility in semicrystalline polymers (*S*) has been assumed to be proportional to the volumetric fraction of the amorphous phase (φ_a_) [70]:

(1) $$S=\phi_{a}S_{a}$$

where *S*_a_ is the compound solubility in the totally amorphous polymer. This physical picture, although successful in many cases and simple to handle, is oversimplified since the sorption behaviour of the amorphous phase is actually affected by the constraints imposed by the presence of crystalline domains. Consequently, the behaviour of the amorphous phase in a semi-crystalline polymer could be generally different from that of a totally amorphous polymer. Besides these constraint effects, the sorption behaviour of amorphous domains strongly depends on the physical state of the polymer: rubbery state (*i.e.*, above glass transition temperature, Tg) or glassy state (*i.e.*, sub-Tg).

The one described above is the behaviour usually displayed by semi-crystalline polymers, however, in few relevant cases (see for example ref [71]) the crystalline domain is characterized by a non-negligible solubility. In particular, sorption studies from liquid and gaseous environments, have shown that nanoporous δ-form and ε-form of s-PS are capable of absorbing a significant amount of suitable guest molecules, even when present at very low concentrations, [68,69,72-84] originating co-crystalline forms [28-35,85].

**Figure 4.**
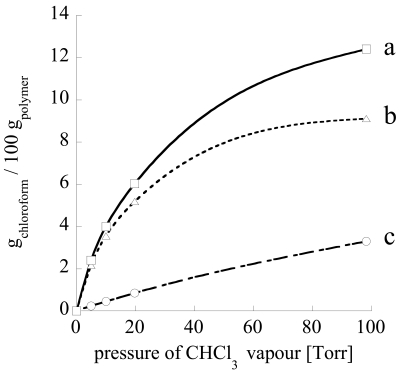
Chloroform vapour sorption isotherms in s-PS δ-form at 35 °C. (a): overall sorption for semi-crystalline sample. (b): calculated contribution of the crystalline phase. (c): calculated contribution of the amorphous phase. Data taken from ref [75]. Lines are drawn to guide the eyes.

**Figure 5.**
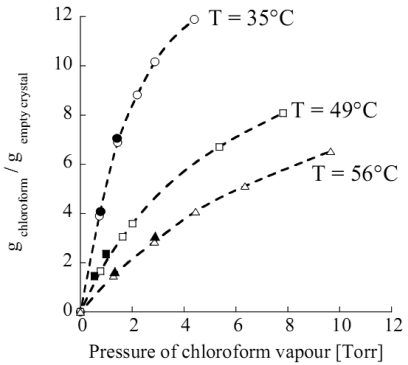
Chloroform sorption isotherms in the crystalline phase of s-PS δ-form films at T = 35, 49 and 56 °C. Lines represent fitting by Langmuir equation. Full marks are simulation values by GCMC. Data taken from reference [84].

As an example, vapour chloroform sorption, performed at 35 °C in semi-crystalline forms of s-PS has shown that in α, β, and γ form samples sorption occurs, in a small amount, only into the amorphous phase. If the relative pressure of chloroform is high enough, α and γ semi-crystalline phases form clathrates while the β semi-crystalline phase remains unaltered [76]. Moreover, totally amorphous samples are reported to readily co-crystallize when exposed to several solvents in vapour phase, including chloroform [19,86,87]. More interestingly, in the case of the nanoporous δ form, a much higher sorption capacity is displayed when the material is in contact with environments where penetrants have a very low activity (here and in the following the term *activity* refers to the *effective* or *thermodynamic* concentration, as by the standard definition available in any general text on physical chemistry, and is commonly used when dealing with non-ideal mixtures. For the case of gas mixtures, if the mixture can be assumed to behave ideally, the activity of component i corresponds numerically to the ratio of partial pressure of component i to its vapor pressure at the temperature of the mixture). This behaviour has been interpreted by considering a relevant sorption contribution of the crystal nanoporous phase. In fact, experimental analyses of chloroform sorption performed using in situ Fourier transform infrared (FTIR) spectroscopy, have definitely confirmed this hypothesis allowing a quantification of the amount sorbed into the amorphous and nanoporous crystalline phases, respectively [75]. As evident in Figure 4, the contribution of sorption in the crystalline phase is dominant in the limit of low pressures (or activies) and tend to level off as pressure is increased, due to progressive saturation of sorption capacity into the nanocavities of the crystalline phase. On this basis, it is expected that the sensitivity of a sensor based on such material, depends upon its sorption capacity in the crystalline phase and changes with the analyte activity. In particular, the sensitivity is the higher the smaller is the analyte pressure (or activity). The amount of analyte sorbed into the crystalline phase is amenable to prediction by using well established techniques of molecular simulation [84], as is reported in Figure 5, where chloroform equilibrium sorbed amount in the crystalline phase of δ-form s-PS, as predicted by Grand Canonical Monte Carlo (GCMC) simulations (filled symbols), compares very well with the experimental data (empty symbols). In the same figure it is shown how the experimental sorption isotherms are well fitted by Langmuir-type sorption isotherms.

**Figure 6.**
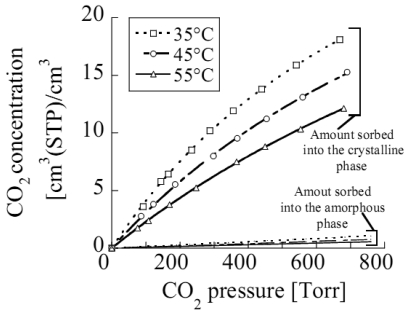
Sorption isotherms of carbon dioxide in the crystalline phase (upper curves) and in amorphous phase (lower curves) of s-PS δ form at several temperatures. Lines represent fitting by Langmuir isotherm (crystalline phase) and Non-Equilibrium Lattice Fluid model for the amorphous phase [83]. Data taken from reference [83].

A relevant sorption capacity of the δ s-PS nanoporous crystalline form has been also reported for other low molecular weight substances [83], e.g., nitrogen, oxygen, carbon dioxide, ethylene and butadiene. In Figure 6, as an example, are reported the sorption isotherms of carbon dioxide for the crystalline phase and the amorphous phase of s-PS δ form.

### 3.2. Guest Diffusivity and Crystalline Phase Orientation

Guest sorption and desorption studies have been conducted for s-PS films presenting the three different kinds of uniplanar orientation of the nanoporous δ phase (*a_∥_ c_∥_, a_∥_ c*_⊥_ and *a*_⊥_ c*_∥_*). These investigations have been performed mainly by using FTIR measurements combined with gravimetric measurements and have been conducted on volatile organic compounds (VOC) like 1,2-dichloroethane (DCE) [67] as well as on gases like carbon dioxide [68] and ethylene [69].

The reported sorption and desorption kinetics data show that, at low guest activities, the guest transport behavior is dependent on the kind of uniplanar orientation of the host crystalline phase. In particular, in agreement with predictions based on molecular simulations [66], the lowest diffusivity has been measured for films with *a_∥_ c_∥_* uniplanar orientation while the highest diffusivity has been measured for films with *a*_⊥_ c*_∥_* uniplanar orientation [67-69]. For application as sensing elements of molecular sensors [88-92], high diffusivity films presenting the *a*_⊥_ c*_∥_* uniplanar orientation would be most suitable, because they maximize the sensor response rates. It is worth of note that analysis of transport of low-molecular-mass compounds in semicrystalline s-PS samples, has evidenced how diffusivities in the amorphous regions are about two order of magnitude higher than any of the diffusivity values in the nanoporous crystalline δ-phase (see, for example, reference [75,83]).

In summary, based on the sorption and diffusion behavior illustrated above, δ form s-PS samples can be considered as a heterogeneous material with a disordered glassy amorphous phase, where the solubility is quite low, and a nanoporous crystalline phase with regularly spaced nanocavities acting as localized sorption sites with well defined dimensions, characterized by a higher sorption capacity. Mass transport of low-molecular-mass compounds in this material can be, hence, envisaged as a process occurring in an heterogeneous medium, characterized by diffusion coefficients which are significantly different in the two coexisting phases. The effective diffusivity of the whole material results hence from the combination of the anisotropic behaviour of the crystalline phase and of the isotropic amorphous phase, characterized by a higher intrinsic diffusivity.

### 3.3. Guest Sorption and Mechanical Properties

A desirable feature of molecular sensing films is the maintenance of their physical state and geometry as a consequence of analyte uptake, possibly over a large number of cycles of use. This is generally a problem for amorphous polymers, which can be plasticized by the sorbed analyte, *i.e.*, can suffer from a substantial reduction of the glass transition temperature (Tg) and rigidity (elastic modulus).

In the case of the illustrated semicrystalline s-PS with a nanoporous crystalline phase, the analyte sorption, mainly at low concentration, preferably occurs inside the crystalline phase, (leading to the formation of the corresponding co-crystalline phase). As a consequence, large analyte uptakes can occur without any change of Tg while the elastic modulus of the semicrystalline film can even slightly increase, due to the increased crystalline phase density produced by the guest sorption [93].

In particular, a careful analysis has been conducted for δ form films (both unstretched and axially stretched) including 1,2-dichloroethane molecules. The choice of DCE was motivated by the additional information, which comes from its conformational equilibrium. In fact, as described in detail in previous papers, [30,67,72,73,77,78] since essentially only its trans conformer is included into the clathrate phase while both trans and gauche conformers are included in the amorphous phase, quantitative evaluation of vibrational peaks associated with these conformers allow to evaluate the amounts of DCE confined as guest in the clathrate phase or simply absorbed in the amorphous phase. The choice of DCE was also motivated by its presence in contaminated aquifers and by its resistance to remediation techniques based on reactive barriers containing Fe^0^ [94,95].

DCE molecules, when are only included as guest into crystalline clathrate phase, have no plasticizing effect. In fact, as a consequence of sorption of 8 wt% of DCE, the Tg of a nanoporous δ film remains unaltered (Tg = 108 °C) while the elastic modulus slightly increases (of nearly 3–5%). In this respect it is worth adding that the DCE equilibrium uptake at room temperature from a δ form sample for 1–100 ppm DCE aqueous solutions is in the range 5–7 wt% [96].

## 4. Gravimetric Sensors

On the basis of the illustrated features, δ-form s-PS films are good candidates as sensing elements for gravimetric sensors aimed at the detection of concentration of analytes (volatile organic compounds) present even at very low activities/pressures in gaseous and aqueous environments. In fact, suitable low-molecular-mass substances can be absorbed both from gaseous as well as liquid environments and penetrate into the material (mainly in their nanoporous crystalline phase), thus determining a weight increase. The rate of weight increase and the maximum equilibrium sorbed amount are respectively ruled by the specific diffusion coefficients and by the equilibrium sorption isotherms.

A simple way to realize such gravimetric sensors, is by depositing a film of δ-form semicrystalline s-PS onto the surface of a quartz disk having metal electrodes (Quartz Crystal Microbalance, QCM). In this way a resonant sensor is obtained whose measuring principle is based on the evaluation of the shift of the quartz crystal resonance frequency which is induced by mass change of the crystal-polymer film assembly, as a consequence of sorption of the analyte into the polymer from the environment. In fact, the natural frequency of the crystal decreases when mass is sorbed into the coating layer. According to Sauerbrey [97] the deposition of a homogeneous coating on an uncoated resonant quartz crystal promotes a shift of frequency that, for a small mass increase (Δ*m*), is described by the following linear relationship:

(2) $$\Delta F=-\frac{2\cdot \Delta m\cdot F_{0}^{2}}{s\cdot A\cdot \sqrt{\mu \cdot \rho }}$$

where Δ*F* is the frequency shift, *F_0_* is the natural oscillation frequency of the crystal, *s* is the harmonic order, *A* is the area of the electrodes on the crystal, *ρ* is the density of the crystal, μ is the elastic transverse force of the crystal and Δ*m* is the mass change due to the deposit. In the case of a coated crystal, the mass change of the sensing film due to sorption of the analyte promotes a depression of the resonance frequency of the system that can be easily monitored. If the thickness of the polymer sensing film is negligible as compared to the quartz thickness and if the film adheres rigidly to the crystal surface and displays proper mechanical properties, the Sauerbrey equation can still be used to relate the mass increase of the total system (quartz and the polymer film) with the frequency shift.

Rubbery polymers can be used as sensing elements for gravimetric sensors, but the sensor response is affected by the viscoelastic behavior of the film when the coating thickness exceeds a threshold value which is material dependent. In this respect, glassy polymers offer the advantage of behaving like a rigid material. Examples relevant in the present context of application of glassy atactic polystyrene as coating on QCM for the detection of volatile organic compounds, both in liquid and atmospheric media, are available in the literature showing good selectivity and sensitivity [98-100]. However, the sensitivity of glassy polymers is often still inadequately low [101,102]. In turn, semicrystalline s-PS δ-form offers the advantage of displaying both a very high sensitivity, related to sorption in the nanocavities of the crystalline phase, and a mechanical rigidity. In fact, since sorption prevalently occurs into the crystalline phase (at least at low to moderate activities), the glassy amorphous phase is still rigid, even in the presence of high amounts of analytes sorbed into the sensing film.

### 4.1. Gravimetric Sensor for Detection of Chloroform in Vapour Phase

**Figure 7.**
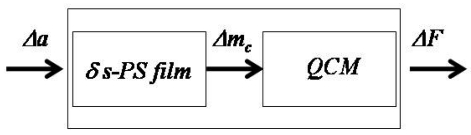
Schematic illustration of the measuring chain for PS coated QCM sensor.

Use of semicrystalline s-PS in δ-form as sensing element for a QCM sensor has been thoroughly discussed in refs [88,103] for detection of chloroform in vapour phase. The schematic architecture of this type of sensor is reported in Figure 7, which shows how the sensor supplies a change in resonance frequency (*ΔF*) as a consequence of mass increase of the sensing layer (*Δm_c_*) due to analyte sorption in response to a step increase of the analyte activity (*Δa*) in the environment (consisting of a vapor phase made only of pure analyte) as obtained by step increasing the analyte pressure. In the following we present the results obtained using a standard AT-cut quartz disk with silver electrodes, with a diameter of nearly 2 cm and thickness of 270 μm, characterized by a main resonance frequency of 6 MHz. To get sensing films, atactic and syndiotactic polystyrene samples have been dissolved into chloroform (1% b.w. solutions) and then cast or spray coated at room temperature on the upper electrode of the quartz disk. The thickness of the films was generally close to 1μm, as evaluated by scanning electron microscopy (SEM). The so obtained s-PS semi-crystalline films were in a clathrate form which was then transformed into the nanoporous δ form after treatment by carbon disulfide vapor at room temperature for 2 hours, followed by desiccation in a vacuum oven (pressure lower than 1 Torr) at 45°C overnight. Details of the experimental set-up are available in refs. [88] and [103].

Tests of the sensor behavior were conducted to monitor chloroform concentration in vapour phase, at several temperatures and pressures, by locating the QCM probe into a water-jacketed stainless-steel chamber operating at a controlled vapor environment.

**Figure 8.**
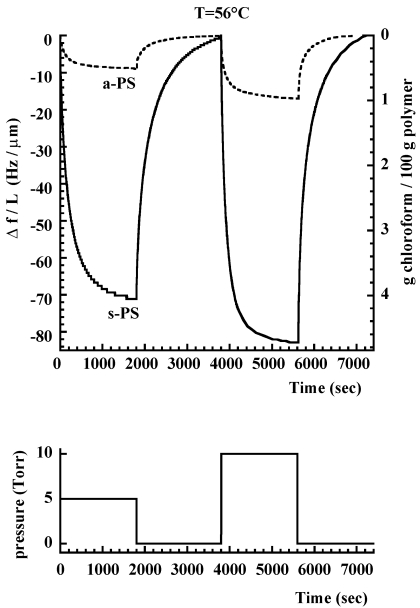
Comparison (upper plot) of the response of a QCM sensor coated by a nanoporous δ form s-PS film (continuous line) with that of a sensor coated by an amorphous a-PS film (dashed line) to square wave changes of chloroform pressure (lower plot). L represents the thickness of coating film. % b.w. increase of the film as a consequence of absorption of chloroform is also reported.

**Figure 9.**
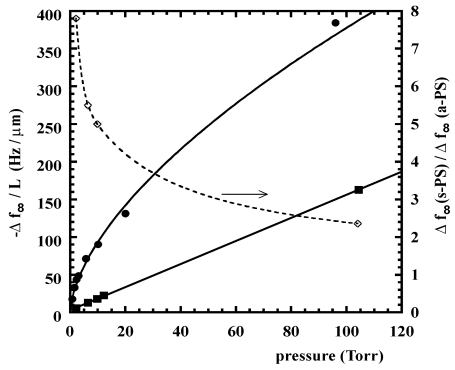
Comparison of the equilibrium response of a QCM sensor coated with a-PS (filled squares) and s-PS (filled circles) as a function of pressure of chloroform vapour. Empty diamonds represent the ratio of sensor outputs (s-PS coated output/a-PS coated output) as a function of pressure.

As an example, in Figure 8 is reported response of a QCM sensor coated with a δ form s-PS film at 56 °C to square wave variations of pressure of chloroform vapor (see lower part of Figure 8) as compared to the response of the same QCM coated with an amorphous a-PS film. The frequency variations obtained after equilibration at different values of chloroform pressure, are compared for the two types of sensors (the one based on a-PS and the one based on a s-PS films) in Figure 9. The ratio between the equilibrium frequency variations for the two sensors is also reported in the same figure. It is apparent that the sensitivity is much larger for the s-PS coated sensor and that this difference remarkably increases as the chloroform pressure decreases (see dotted line in Figure 9).

The observed behavior remarks again the high sensitivity attainable due to high sorption capacity of low m.w. molecules in the nanoporous crystalline phase of s-PS which, in turn, determines a large mass increase of sensing film when exposed to analyte vapor, even at very low activities.

**Figure 10.**
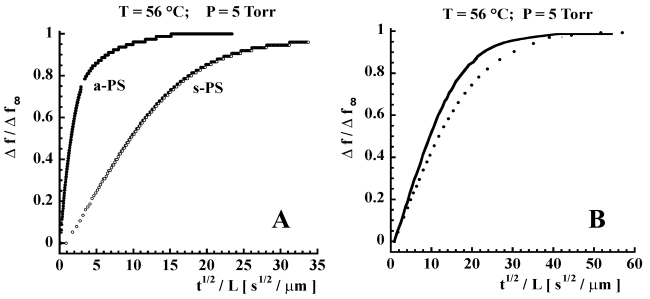
(A) Comparison of the response of QCM sensor for an a-PS and s-PS sensing layers. Data obtained at 56 °C and at a chloroform pressure of 5 Torr. (B) Reversibility of the sensor response.

Although δ-form s-PS ensures a much higher sensitivity as compared to amorphous a-PS, this material is characterized by lower diffusivities which determine slower response times. This is evident from Figure 10a where are compared the kinetics of a-PS and s-PS based sensors, in response to an instantaneous increase of chloroform pressure (from zero to 5 Torr, at 56 °C). However, the response rate of the s-PS based sensor could be increased, without loosing sensitivity, by changing in several ways the macroscopic and/or microscopic structure of the sensing material. In fact, a faster response can be obtained by increasing the specific surface through foaming of the polymer layer (e.g., by using aerogels exhibiting nanoporous crystalline phases) [53,79], a process which decreases the effective thickness of the sample, but not its total mass. Another approach could be to change the orientation of the crystalline phase. Actually the samples used in the investigation reported in refs [88,103], are characterized by a uniplanar orientation *a_∥_ c_∥_* of the crystalline phase, that is induced by the spin-coating process. This kind of orientation leads to a low diffusivity of the analyte, in the direction perpendicular to the film itself, due to the low inter-chain distances (0.87 nm). Since the crystalline phase is characterized by anisotropy of the diffusion coefficient (see discussion in Section 3.1), a substantial increase of rate of sorption into the film could be obtained by orienting the δ-form crystallites with their highest diffusivity direction (the *a* axis) perpendicular to the film plane. (*i.e.*, by using films exhibiting the *a*_⊥_ *c_∥_* uniplanar orientation, see Section 2.4).

The good reversibility of s-PS sensor is witnessed by sorption-desorption data (pressure change from 0 to 5 Torr and from % to 0 Torr, T = 56 °C) reported in Figure 10b. It is, also, apparent that guest desorption is significantly slower than guest absorption: this behavior can be easily rationalized by considering the concentration dependence of chloroform diffusivity [75].

The peculiar sorption behavior in the crystalline phase, which plays a major role in absorption process at low activities, gives potential advantage also in terms of selectivity. Actually, the formation of host-guest compounds is intrinsically more selective than the mechanisms associated to sorption in amorphous polymers. Molecules larger than the cavities of the nanoporous crystalline form of s-PS (like, e.g., chlorodecane, chloronaphtalene) are excluded from the crystalline domains and can be sorbed only into the amorphous phase of semicrystalline s-PS, leading to much lower sorption levels. For those bulkier molecules, the response of the semicrystalline s-PS sensor is expected to be even smaller than in the case of an a-PS based sensor (in fact, for the investigated samples the amorphous phase is about 60% of total mass).

### 4.2. Gravimetric Sensor for Detection of Chloroform in Liquid Water

Direct measurement of concentration of organic halogenated compounds is of great interest both for evaluation of water potability and for the detection of their presence in industrial discharge streams. In the first case the maximum contaminant level admissible, in the case of presence of chloroform as the only halogenated pollutant, is in the order of few tens of ppm. QCMs coated with nanoporous semi-crystalline s-PS films prove to give good results in terms of sensitivity and repeatability also for detection of halogenated compounds in water streams. The disadvantage of s-PS polymeric sensitive layers is still a limited selectivity (as compared, for example, to calixarenes) counterbalanced by a greater reversibility, which is instead a weak point for more selective materials.

The results reported in the following were obtained using quartz crystals of AT cut type produced by Maxtek (model 149211-2/149238-2) with a resonance frequency in the range 4,975–5,020 MHz with a sensitivity of 0.056 Hz/(ng cm^2^) at 20 °C. The surface finishing was of the type ‘unpolished’ and the electrodes were of the gold/gold-titanium type. The coating procedure was similar to the case of vapour phase measurement. Crystals were housed in a CHK-100 retainer inserted in a FC-500 Flow Cell assembly and exposed to a continuously flowing water stream containing chloroform at a known concentration. The instrument for the measurement of resonance frequency of the coated crystal was an RQCM from Maxtek. To insure a higher accuracy of resonance frequency measurement in case of high damping (as is the case when the crystal is in contact with high viscosity fluids) the RQCM keeps trace also of the crystal electric resistance.

Step changes of chloroform concentration in the water stream were realized by introducing known amounts of the analyte using a precision microsyringe. Frequency shift of the crystals was continuously monitored and when a steady state was reached another step increment of concentration of chloroform was realized by injecting some more known amount of contaminant into the reservoir.

**Figure 11.**
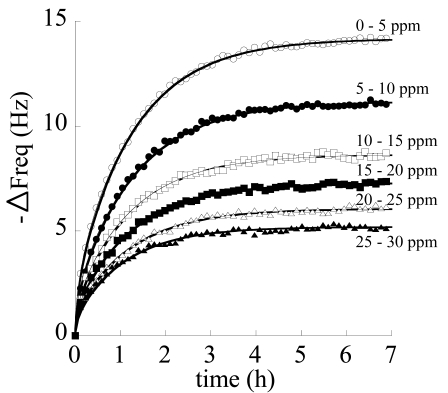
Change in frequency as a function of time in response to step changes in chloroform concentration. Solid lines represent fitting of data with Equation 4.

A typical experiment, conducted at 30°C, on a crystal coated with a 5 μm s-PS δ form film, consisted in realizing a final concentration of chloroform in the circulating stream of 30 ppm, obtained by successive 5 ppm step increments of concentration. In Figure 11 are reported, collectively, all the results of the step sorption tests conducted in a typical run where step increases of 5 ppm (*i.e.*, 0-5-10-15-20-25-30 ppm) in concentration are successively realized. Continuous lines were obtained by fitting data with a model obtained by solving a one-dimensional differential mass balance on the penetrant (chloroform) describing the sorption process into the film and adopting a Fick's law as constitutive equation for mass flux. In fact, the following expression:

(3) $$M\left(t\right)=M_{\infty }\left\{1-\sum_{n=0}^{\infty }\frac{8}{{\left(2n+1\right)}^{2}\pi^{2}}\exp\left[-{\left(2n+1\right)}^{2}\pi^{2}\frac{\mathrm{Dt}}{4l^{2}}\right]\right\}$$

describes [104] the evolution with time of the integral amount of penetrant mass (M(t)) sorbed into the deposited film of thickness *l*, assuming a one-dimensional geometry. The quantity, *M*_∞_, represents the amount sorbed at equilibrium, *D* is the penetrant diffusivity (fitting parameter) and *t* is the time. From Equation 3, the expression for the evolution with time of frequency shift is readily obtained in view of Equation 2. In fact a simple proportionality relationship holds between sorbed mass of penetrant and induced frequency shift. Consequently, the following equation has been used to fit data:

(4) $$\Delta F\left(t\right)=\Delta F_{\infty }\left\{1-\sum_{n=0}^{\infty }\frac{8}{{\left(2n+1\right)}^{2}\pi^{2}}\exp\left[-{\left(2n+1\right)}^{2}\pi^{2}\frac{\mathrm{Dt}}{4l^{2}}\right]\right\}$$

Here Δ*F*_∞_ represents the equilibrium frequency shift realized in response to each step increase in chloroform concentration in the aqueous solution in contact with the sensing layer. As is evident in Figure 11, a very good fitting of kinetic response of the sensor to successive step increases in chloroform concentration is obtained with Equation 4.

**Figure 12.**
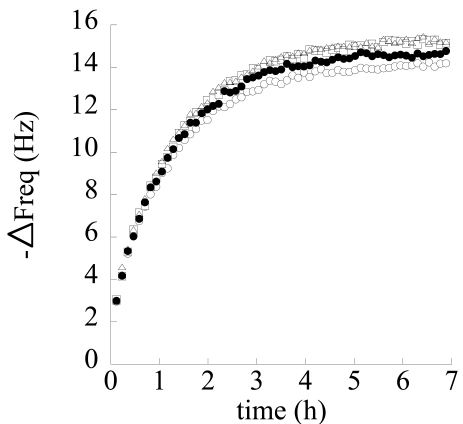
Repeatability tests for the 0–5 ppm step change in chloroform concentration.

Runs were repeated several times on the same samples. Typical repeatability results referred to the 0–5 ppm step test are reported in Figure 12.

**Figure 13.**
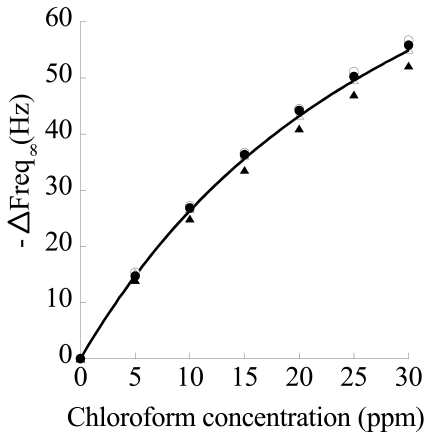
Plot of static response of the sensor as a function of chloroform concentration. Solid line represents data fitting with Equation 5.

The steady state responses of the sensor as a function of concentration in the different runs are reported in Figure 13. Data are well fitted by a Langmuir type expression for frequency shift:

(5) $$\Delta F=\frac{a\cdot c}{1+b\cdot c}$$

This result is supported by the physically sound hypothesis that sorption mainly occurs in the nanoporous crystalline phase and is ruled by an adsorption mechanism where each adsorption site (crystalline nanocavity) is able to host just one molecule. In Table 1 are reported average values of equilibrium frequency shift, along with their standard deviation, for the performed tests.

**Figure 14.**
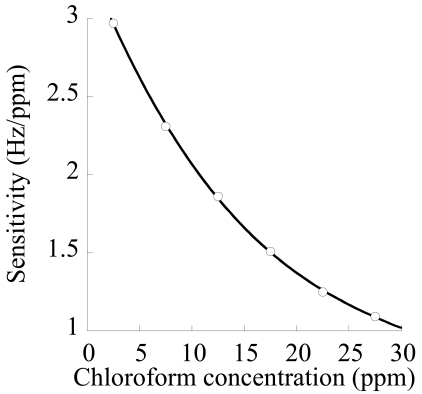
Plot of experimental and theoretical sensitivity (solid line) as a function of chloroform concentration in aqueous solution.

Finally, the theoretical sensitivity (evaluated as the derivative of ΔF as a function of concentration, from Equation 5) *vs.* chloroform concentration is reported in Figure 14, along with experimental data (evaluated as the ratio of frequency shift and concentration increase in each step). As already discussed, sensitivity decreases as concentration increases. Overall, gravimetric sensors coated with nanoporous semi-crystalline s-PS display a good sensitivity and repeatability for detection of chloroform concentration also in aqueous streams and the results are amenable to a physically sound interpretation based on the available model for crystalline structure.

## 5. Fiber-Optic Sensors

Nanometric thin films of s-PS in the empty nanoporous *δ* form have been also successfully integrated with fiber optic technology for the realization of chemical sensors able to detect few parts per million VOCs pollutants both in air and water environments.

Two types of fiber optic sensing platforms were exploited both based on the refractive index change of the sensitive s-PS layer upon analyte sorption. The first one makes use of a cleaved fiber optic ended with an s-PS thin film interrogated in reflectometric configuration. The second one exploits Long Period Gratings whose cladding is overlayered by thin films of s-PS.

### 5.1. Reflectometric Configuration

**Figure 15.**
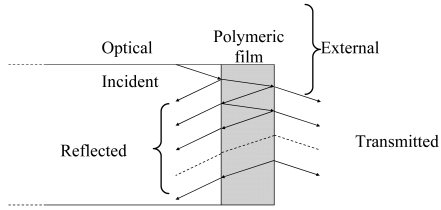
Schematic of the multiple beams interference in a F-P cavity formed by a thin layer of s-PS deposited on a cleaved fiber optic. Beams angles are exaggerated for explanatory reasons.

When a thin polymeric film is deposited onto the distal end of a cleaved optical fiber a Fabry-Perot cavity is formed and the amount of light power reflected back from the fiber tip is the result of multiple beams interference within the cavity (see Figure 15).

In this configuration the reflectivity is given by the formula [105]:

(6) $$R=\frac{{\left(r_{12}+r_{23}\right)}^{2}-4r_{12}r_{23}\sin^{2}\left(\delta \right)}{{\left(1+r_{12}r_{23}\right)}^{2}-4r_{12}r_{23}\sin^{2}\left(\delta \right)}$$

with:

$$\begin{array}{ccc} r_{12}=\frac{n_{f}-n_{p}}{n_{f}+n_{p}} & r_{23}=\frac{n_{p}-n_{\mathrm{ext}}}{n_{p}+n_{\mathrm{ext}}} & \delta =\frac{2\pi \mathrm{nd}}{\lambda } \end{array}$$

where *r_12_, r_23_* are the Fresnel reflection coefficients for normal incidence at the fiber–polymer and polymer—external medium interfaces, respectively, *δ* is the phase shift that the light of wavelength *λ* undergoes when it passes through the polymer layer of thickness *d; n_f_*, *n_p_, n_ext_* are the refractive indices of the fiber core, the polymeric layer and the external medium respectively. As it can be clearly inferred by this formula the reflectivity depends on the optical thickness *nd* of the polymeric layer and therefore a change of its refractive index *n* and/or thickness *d* produces a change of the reflectivity *R*. However it was already highlighted that at low concentrations of analyte the absorption occurs in the crystalline phase of the s-PS without a thickness change but rather with a refractive index change of the layer.

**Figure 16.**
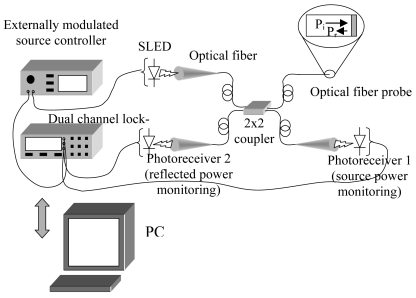
Schematic of the reflectometric interrogation system.

The interrogation system of the fiber probe is depicted in Figure 16 and its detailed description can be found in [106]. It comprises a superluminescent light diode (SLED) whose power is split through a 2 × 2 coupler in two channels, one directed to the cleaved fiber optic with the sensitive layer, the other one to a photodiode that returns a signal proportional to the source intensity and which is needed to compensate its power fluctuations. The light power reflected back from the fiber tip is monitored by another photodiode. The optoelectronic sensor output *I*, consists of the ratio between the reflected signal and the one corresponding to the power monitoring and it results to be proportional to interface reflectance *R* that is *I* = *αR*, where *α* takes into account some set-up parameters. Improved signal to noise ratio is obtained with synchronous detection by using a dual channel lock in amplifier.

The expression of the reflectance and hence of the sensor output can be linearized with respect to small refractive index changes around the s-PS refractive index *n_p_* in the assumption that no thickness change occurs to the polymeric layer:

(7) $$R={R_{0}(d,n_{p})+\frac{\partial R}{\partial n}|}_{d,n_{p}}\left(n-n_{p}\right)$$

therefore:

(8) $$\Delta n=K_{n}\Delta I$$

In this way it is possible to show that by increasing the overlay thickness the sensitivity of the sensor increases [107]. Unfortunately also the sensors response time *θ* increases by increasing the layer thickness (*θ ∼ d^2^/D* for polymer-penetrant systems following a Fickian behavior, where *d* is the thickness of the sensitive polymer film and *D* the diffusivity of the chloroform within the polymer). Moreover, Equation 8 establishes a linear relationship between the sensor output change and the layer refractive index change once the calibration constant *K_n_* is known.

For what concerns the sensor preparation, the acrylic jacket of a standard monomode optical fiber SMF-28 was accurately removed and the fiber was cut with a precision cleaver in order to obtain a plane face orthogonal to the light propagation direction along the fiber axis. The fiber optic tip was then dip-coated in an s-PS (1.5% b.w) solution in chloroform. In this way a clathrate thin film of s-PS was formed at the distal end of the fiber. The film was finally exposed to acetone vapors in order to extract the solvent and obtain the empty nanoporous δ form of the semi-crystalline s-PS. The crystalline fraction of the δ form obtained by a casting from a chloroform solution is about the 50% by volume [38] and presents a strongly uniplanar orientation, in other words the ac planes are parallel to the sensor surface [54].

The measurement of the s-PS refractive index was performed by an interferometric technique based on wavelength dependent reflectance measurements around 1,310 nm [107]. In particular the ratio between the reflected spectrum from the coated fiber tip and from the bare cleaved fiber was considered for films of different thickness. The obtained experimental values could be fitted with the reflectivity formula. The s-PS refractive index was found to be approximately 1.578 which is consistent with data obtained by using a commercial Abbe refractometer [55]. The same method was also used to estimate the thermo-optic coefficient of the sensing overlay, which was found to be approximately −4.3 × 10^−4^ K^−1^.

On the basis of the aforementioned considerations, the optimization of the thickness of the sensitive layer was based on a trade-off between a fast response and a good resolution of the measure. In particular, a standard silica optical fiber coated with a 73 nm thin film of s-PS in the nanoporous *δ* form was used for the detection of chloroform in the vapor phase at three different temperatures: 35, 49 and 56 °C.

Reflectometric measurements consisted in recording the sensor output as the sorption of the analyte in the nanocavities promoted a change of the polymer layer refractive index. In particular, the correlation between the refractive index changes and the chloroform concentration within the sensitive film can be described by the Lorenz-Lorents law [108]:

(9) $$\frac{n^{2}-1}{n^{2}+2}=\frac{N}{3Mɛ}\rho \beta$$

where *ρ* is the density, *β* the polarizability, *N* the Avogadro number, *M* molecular weight of the repetitive polymer unit and *ε* the vacuum permittivity. Hence, the refractive index change is determined by the combined variation of the density and of the polarizability, which is clearly affected by specific interactions between the chloroform molecules and the cavities walls.

**Figure 17.**
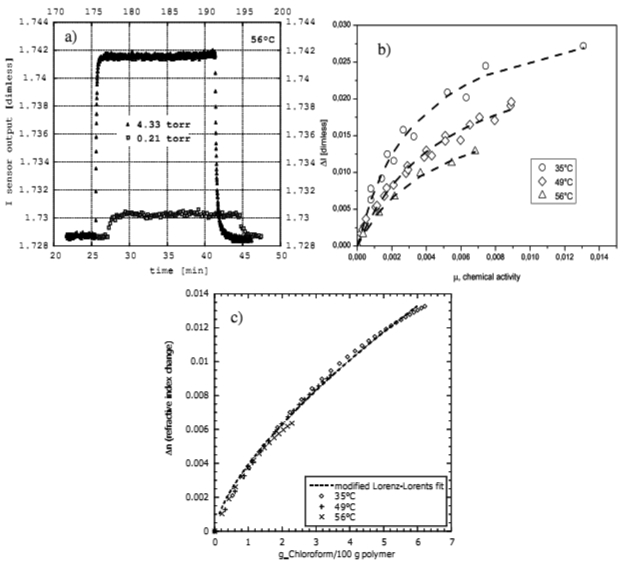
(a) Typical step responses of the s-PS coated fiber optic tip in the reflectometric configuration for different pressures of chloroform in vapor phase at 56 °C; (b) reflectometric sorption curves at different temperatures; (c) relationship between refractive index change of sPS and absorbed mass of chloroform.

**Figure 18.**
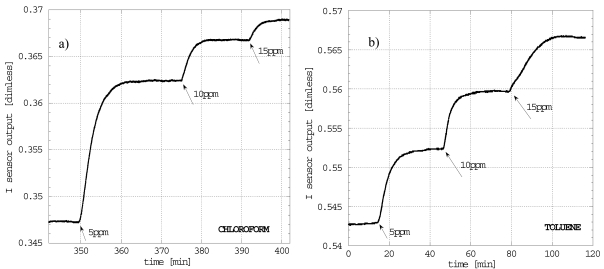
(a) Sorption curve for chloroform in water at 25 °C. Each step corresponds to a 5 ppm addition of solute in water;(b) Toluene sorption curve in the same test conditions of (a).

The typical step responses of the fiber optic sensor upon sorption of an analyte are shown in Figure 18a, in particular the diagram reports the response to an instantaneous increase of the chloroform pressure from zero to 0.21 Torr and to 4.33 Torr at 56 °C, where the response times are 16 and 14 sec. respectively. The sensor output at equilibrium at each temperature was then reported as a function of the chloroform vapor activity (expressed as the ratio between the pressure of chloroform, p, and its vapor pressure, p_0_, assuming an ideal behavior of the vapor phase) to obtain isotherm reflectometric curves (see Figure 17b). The linearized expression in Equation 8 allowed direct correlation between the output signal variations and the sensing polymer film refractive index variations.

The calibration constant *K_n_* for the considered probe, *Δn/ΔI*, was found to be 0.493. Calibration was experimentally performed by using the previously characterized thermo-optic coefficient and the change of the sensor output with the temperature. The resolution of the used optical sensor, expressed in term of minimum refractive index variation detectable, was 4.9 × 10^−5^.

Gravimetric sorption tests were performed by means of an electronic microbalance (Cahn D200) on s-PS powder samples of 10 nm average diameter in the same conditions of temperature and pressure as for the fiber probe. The s-PS samples were prepared by milling films of the polymer obtained by casting from chloroform solution, exposed to acetone and dried in air at room temperature. A full description of the experiment can be found in reference [107]. However, what is important to recall here is that the data thus collected provided a quantitative correlation among the chloroform concentration in the polymer upon sorption, the corresponding change of the polymer refractive index, and the external pressure of chloroform.

Figure 17c shows the refractive index change occurring at the sPS sensitive film as a function of the adsorbed mass of chloroform for the three different opertating temperatures. The experimental data are well fitted with a semi-empirical equation derived by the Lorenz-Lorents law [108] and this relationship is almost independent on the temperature. However the most interesting thing is the impressive refractive index change of the order of 10^-2^ for a few grams of chloroform absorbed each 100 grams of polymer, which is obtained for activities of about 6 × 10^−3^ at 35 °C. This demonstrates a high sensitivity of the material itself at very low concentration of analyte.

The cleaved fiber tip coated with a thin film of s-PS in the empty δ form was also used to detect trace amounts of two organic analytes, chloroform and toluene, in an aqueous environment [90]. The deposition procedure of the sensitive layer onto the tip of the standard silica fiber was already illustrated. The sensor head was inserted in a thermostated baker (25 °C) containing initially pure distilled water.

Again, reflectometric measurements consisted in recording the sensor output changes due to a change of the polymer layer refractive index promoted by the analyte sorption. In this case, analytes were injected into the test environment by successive steps of 5 ppm (μl/l) up to 15 ppm while the solution was continuously stirred to ensure the maximum dispersion of the analyte. Moreover the sensor reversibility was verified after each absorption test by washing the stirred solution with a flux of distilled water until the desorption process reached the plateau.

In order to have a comparison with another transducer, similar measurements were carried out with a QCM whose surface was spin coated with an sPS film of about 500 nm. Figures 18a and 18b show selected reflectometric sensor responses obtained with a layer of about 200 nm thickness.

The fiber optic response to the addition of analyte is almost instantaneous after the injection and the equilibrium plateau is reached in few minutes. Response time depends obviously on the thickness of the coating film: the thinner is the layer the faster is the attainment of the response equilibrium. In the case of chloroform the response time of the sensor to the first step of 5 ppm was about 6.5 min, while the ΔI was about 0.015. It is worth noting that the sensitivity of the sensor progressively decreases for successive steps along with the response time. In particular a ΔI of about 5 × 10^−3^ with a response time of 3.75 min was observed in the range 5-10 ppm, and 2 × 10^−3^ with a response time of 3.8 min in the range 10–15 ppm.

The reason of this behaviour, for what concerns the equilibrium response, is related to the non linear relationship between absorbed mass of chloroform and its concentration in the liquid phase, while the change of the response time is due to the change of the diffusivity with the concentration of the absorbed analyte [75]. For the toluene the decrease in sensitivity is less pronounced likely due to the fact that, for the investigated concentrations, toluene sorption equilibrium is described by a linear relationship. In fact the sensitivity is almost constant in the range 0–15 ppm with a ΔI of about 9 × 10^−3^ for a single 5 ppm injection. Also the response time reduction, from 9.7 min to 7.4 min for the first 2 steps, was less remarkable. Even if the s-PS is not able to discriminate between two organic analytes, it is worth noting that the chloroform kinetic is faster than that of toluene and this suggests a possible discriminative property between the two VOCs. Moreover, to analyze the reproducibility of the response, four different sensors were prepared by dip-coating the same s-PS chloroform solution (1.5% by weight) on four different fibers.

A linearized and normalized expression of the reflectivity allowed direct comparison among the equilibrium responses of fiber optic sensors with different coating film thickness showing good reproducibility of the measure of the sorption induced refractive index change.

The reversibility of the prepared sensors was, then, verified by monitoring for each sensor the complete cycle of sorption of the analyte followed by a washing with pure water flux. From the comparison with the QCM sensor, the main results are that, in the cases of the studied analytes, the optoelectronic based sensor displays a better resolution in spite of the lower thickness of the sensing layer. In turn this finding assures a faster response of the optoelectronic sensor in respect to the QCM sensor [90].

### 5.2. s-PS Coated Long Period Gratings

**Figure 19.**
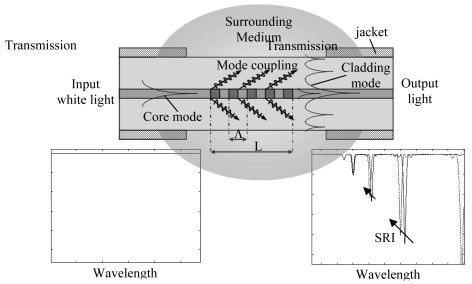
Pictorial description of mode coupling in LPGs and spectral dependence on SRI (not to scale).

Long Period Fiber Gratings (LPFGs or LPGs) are in-fiber diffraction gratings realized by inducing a periodic refractive index modulation (typ. 100–500 μm period) of the core of a single mode optical fiber along few centimeters of its length (typ. 2–3 cm). LPGs act coupling the fundamental guided core mode to discrete forward propagating cladding modes and to each of them at a distinct wavelength *λ_res,0i_* where the so-called phase matching condition is satisfied [109]:

(10) $$\lambda_{\mathrm{res},0i}=\left(n_{\mathrm{eff},\mathrm{co}}-n_{\mathrm{eff},\mathrm{cl}}^{0i}\right)\cdot \Lambda$$

where *_n_eff,co* and $n_{\mathrm{eff},\mathrm{cl}}^{0i}$ are the core and *i^th^* cladding mode effective indices respectively, *Λ* is the grating period. As a result of the mode coupling process the LPG transmission spectrum shows several attenuation bands or dips related to the different excited cladding modes (see Figure 19).

LPGs are sensitive to a number of environmental parameters (temperature, strain, bending, external refractive index) which affect the phase matching condition changing, in turn, the attenuation bands spectral position. However the dependence of the LPG spectral features on the surrounding medium refractive index (SRI) changes is what makes them particularly attractive for chemical sensing applications [110].

The effective refractive indices of the cladding modes increases by increasing the SRI and therefore the attenuation bands shift to shorter wavelengths as a consequence of such SRI increase. The spectral displacement of the attenuation bands can be used to measure the SRI changes. This is true for SRIs smaller than the cladding refractive index, in fact when the SRI matches the cladding refractive index the core mode is coupled to radiation modes and attenuation bands disappears.

The higher the cladding mode order, the higher the wavelength shift. This is because the higher order cladding modes extend further into the surrounding medium with their evanescent tail and hence are more sensitive to SRI changes.

**Figure 20.**
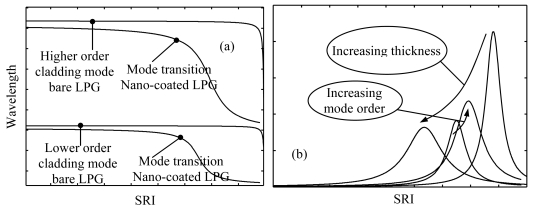
Conceptual description of the modal transition in nano-coated LPGs; (a) comparison of the dips shift behavior in the bare device and in the coated one; (b) sensitivity characteristics in the coated device for fixed cladding mode and varying overlay thickness.

The sensitivity characteristic of a bare LPG to SRI changes has an increasing non-linear monotone trend. The result is that the maximum sensitivity is achieved for SRIs close to that of the cladding while for SRIs around the water refractive index the LPG is much less sensitive (see Figure 20a).

The situation is different when a thin layer of sub-wavelength thickness (ranging in hundreds of nanometers) and with higher refractive index (HRI) than the cladding is deposited onto the latter. The HRI overlay draws the optical field towards the external medium extending its evanescent wave. As a result there is an increased sensitivity of the device to the SRI changes [111].

Moreover the HRI overlay is a wave guide itself and allows mode propagation depending on its thickness, refractive index (RI) and on the SRI. For a given material (fixed RI) and overlay thickness, when the SRI is varied in a certain range the lowest order cladding mode is gradually and completely sucked into the overlay and becomes an overlay mode. At the same time all the higher order cladding modes effective indices shift to recover the previous effective indices distribution. This is reflected through the phase matching condition in the shift of the attenuation bands toward the next lower one (see Figure 20a). In the middle of this modal transition the attenuation bands can exhibit sensitivity (|∂λ_res_/∂SRI|) of thousands of nanometers per unitary change of SRI. The sensitivity characteristic of the coated LPG is drastically modified compared to the bare device [112]. It has a resonant-like shape whose resonant peak can be tuned around the desired SRI by changing the overlay thickness (see Figure 20b).

The trend of the sensitivity to SRI changes in the coated device goes hand in hand with that of the sensitivity to the refractive index changes of the polymeric overlay. This is justified by the fact that an increasing evanescent wave in the surrounding medium corresponds to an increasing optical power fraction confined in the overlay. Moreover the sensitivity to the refractive index changes of the polymeric overlay is also bigger than that to the SRI changes because of a more powerful guided wave interaction than an evanescent wave interaction [92].

From a chemical sensing perspective, the HRI overlay can be a chemo-sensitive material able to specifically collect and concentrate in its volume molecules from the surrounding environment undergoing a consequent refractive index change as in the case of the s-PS in the empty delta form.

The sensor design strategy consists in optimizing the sensitivity of the transducer with a layer thickness such that the cladding modes are in the transition region for the specific refractive index of the medium where the chemical detection has to be performed obtaining at the same time optimized sensitivity to the overlay refractive index changes.

It is worth to note that the injection of few ppm of an analyte in the test environment causes negligible changes of the SRI compared to the absorption induced refractive index changes in the sensitive layer.

Thin polymeric films can be deposited onto the silica cladding of an optical fiber by means of the dip-coating technique. This technique consists in immersing the fiber-substrate in a liquid solution and then in withdrawing it with well controlled extraction speed.

The film thickness depends upon many parameters such as the withdrawal speed, the solid content and the viscosity of the liquid. If the withdrawal speed is chosen such that the shear rates keep the system in the Newtonian regime, then the coating thickness depends upon the aforementioned parameters by the Landau-Levich equation [113]:


(11) $$\mathrm{th}=0.94\cdot \frac{{\left(\eta \cdot \nu \right)}^{\overset{2}{\;}/\underset{3}{\;}}}{\gamma_{LV}\cdot {\left(\rho \cdot g\right)}^{\overset{1}{\;}/\underset{6}{\;}}}$$

where *th* is the coating thickness, *η* the solution viscosity, *ρ* the density, *γ_LV_* the liquid-vapor surface tension, *g* the gravity and *v* is the withdrawal speed.

In order to apply the dip-coating technique to the grating region along the optical fiber a special holder was designed and realized. In particular the holder ensures that the LPG is kept straight and in similar tensional state conditions both during the coating procedure and during further sensor testing in order to minimize the effects of the LPG cross-sensitivity to other environmental parameters.

**Figure 21.**
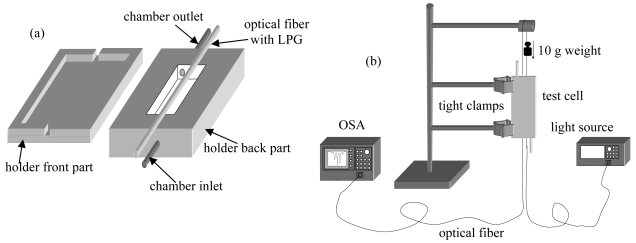
(a) Schematic of the LPG holder for thin film deposition by dip coating and for analyte testing; (b) Schematic of the opto-electronic set-up used for the interrogation of LPGs.

The holder comprises two complementary moulds completely realized in Teflon, forming the test chamber as shown in Figure 21a. Here, a V-groove geometry allows a good placement of the optical fiber. The optical fiber with the LPG can be arranged in the holder and put in vertical position as illustrated in Figure 21b. A small weight acting on the fiber by a pulley ensures a similar strain on the LPG in different experiments.

The optoelectronic set-up, involved for sensor fabrication monitoring and for further sensor testing (see Figure 21b) comprises two broadband superluminescent diodes operating at 1,310 nm and 1,550 nm, respectively, and an optical spectrum analyzer for transmitted spectrum monitoring.

The LPG employed in the experiments was a commercial 30 mm long LPG, with a nominal period of 340 μm, written in a standard Corning SMF-28 optical fiber.

Thin films of sPS can be deposited onto the LPG by filling up the chamber by means of a syringe with a solution of s-PS in chloroform and then emptying it out in few seconds. Different overlay thickness can be obtained by different extraction speeds and/or solution viscosities. As already discussed, just after the deposition a suitable procedure was applied to facilitate the solvent evaporation. A first overlay deposited onto the LPG was found to be approximately 160 *nm* (*th_1_*).

**Figure 22.**
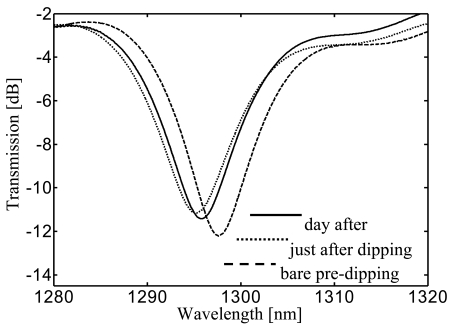
Shift of the attenuation band related to the cladding mode LP_06_ due to the deposition of a 160 nm s-PS overlay.

Figure 22 shows a comparison between the transmitted spectra of the bare and the 160 *nm* s-PS coated LPG, with regard to the cladding mode LP_06_. Two effects are evident just after the dipping: a blue shift of *λ_res_* of 2.45 *nm* and a decrease of the transmission loss peak of about 1 *dB*. After the complete desorption of the solvent molecules from the polymeric layer, the transmission spectrum demonstrated a final blue shift of 1.8 *nm* and a decrease of the transmission loss peak of 0.8 *dB* compared to the bare one.

The HRI coating induces an increase in the effective refractive index of the cladding modes and thus a decrease in the resonance wavelengths. In addition, the spatial shift of the cladding mode field profile toward the HRI overlay promotes a decrease of the overlap integral with the core mode and so of the transmission loss peak. The subsequent evaporation of the solvent molecules from the nano-cavities results in a reduction of its refractive index. This explains the partial recovery of the attenuation band initial position. Again a blue shift of the attenuation band is expected during exposure to analyte since chemical absorption within the polymeric layer induces an increase in its refractive index.

In order to test the sensor response to overlay RI changes induced by chemical sorption, chloroform was used as analyte. A similar holder to that used for the deposition was used to host the coated LPG allowing also the conveying of pure distilled water or polluted water as the case. The temperature was held constant at 20 °C.

It is worth noting that for the considered overlay thickness and for an SRI = 1.33 the cladding modes were still far from the transition region, so that a moderate sensitivity of the device was expected.

**Figure 23.**
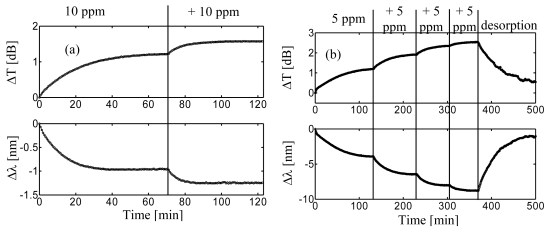
(a) Attenuation band transmittivity and central wavelength changes during analyte sorption for a 160 nm coated LPG. The attenuation band is related to the cladding mode LP_06_ and is placed in a spectral window around 1,310 nm; (b) attenuation band transmittivity and central wavelength changes during analyte sorption for a 260 nm coated LPG. The attenuation band is related to the cladding mode LP_08_ and is placed around 1,550 nm.

Measurements consisted in recording the transmission spectra of the sensing grating as the sorption of the analyte in the nanocavities promoted an increase of the polymer layer refractive index. The spectra were recorded each 40 seconds. The holder, with the s-PS coated LPG, was connected with a thermostated beaker (20 °C), containing initially 1 liter of pure distilled water. In this way, the holder was filled up of pure water. Chloroform was then added in successive steps of 10 ppm (μL/L). The solutions were always magnetically stirred in order to ensure the maximum dispersion of the analyte in water and then fluxed to the sensor holder. Figure 23a shows the time responses of the sensor in terms of wavelength shift and amplitude changes of the considered attenuation band to two successive 10 ppm chloroform exposures [92].

At these concentration levels negligible effect on the SRI occur, thus changes in the attenuation bands here reported can be attributable only to the chemical sorption within the sensitive overlay. The 10 ppm and 20 ppm chloroform concentrations induced a blue wavelength shift of 0.96 nm and 1.26 nm, respectively, and a decrease of the loss peak of 1.20 dB and 1.57 dB.

Here, a response time (10%–90%) of about 21 minutes (*τ_1_*) for 10 ppm chloroform concentration was measured. As the dynamic of the chemical sorption relies on the diffusion of the analyte through the sensitive overlay, the overlay thickness drives the time response of the sensor. The thinner the overlay, the faster the sensor response.

Moreover, the dependence of the response times on the analyte concentration and the decrease of the sensor outputs was already discussed for the reflectometric configuration.

In order to exploit the sensitivity enhancement obtained when the transition region is approached, the first overlay was removed and a new thicker overlay was deposited using the same technique. Here, the measured thickness was approximately 260 nm (*th_2_*) and an attenuation band was found in the 1,550 nm spectral region with water as surrounding medium. This attenuation band was originally placed around 1,700 nm when the overlay thickness was 160 nm, but the new overlay thickness was big enough to tune the attenuation band (with SRI = 1.33) in a useful spectral window to be interrogated (around 1,550 nm) and where it could considered in the middle of its modal transition. Therefore an higher sensitivity of the device was expected in this case due to both the higher order mode considered and to the sensitivity enhancement offered by the modal transition.

In order to prove the sensitivity enhancement when the attenuation band works within the transition region, a similar experiment to the one described above was carried out.

Figure 23.b shows the time responses of the sensor in terms of wavelength shift and loss peak changes due to four successive 5 ppm chloroform exposures. A cumulative blue wavelength shift of 3.95 nm, 6.45 nm, 8.03 nm, 8.79 nm and a decrease of the transmission loss 1.20 dB, 1.91 dB, 2.34 dB, 2.53 dB, were measured. In this case, the response time (10–90%) was estimated to be about 62 minutes (*τ_2_*) for a single 10 ppm step (not reported here).

When chloroform was removed by enabling a continuous flux of distilled water, an excellent recovery was observed demonstrating the reversibility of the sensor.

With regard to the response times, according to the diffusion theory and to the experimental results previously reported a quadratic rule can be assumed [107]. This means that the thickness ratio matches the square root of the response times ratio:

(12) $$th_{1}/th_{2}={\left(\tau_{1}/\tau_{2}\right)}^{0.5}$$

where *th_1_* and *th_2_* are overlay thicknesses and *τ_1_* and *τ_2_* are response times. In our case, with *th_1_* ≈ 160 nm *th_2_* ≈ 260 nm, *τ_1_* = 21 minutes and *τ_2_* = 62 minutes, we obtain 0.615 for the left side and 0.586 for the right one demonstrating good agreement with the data previously reported.

**Figure 24.**
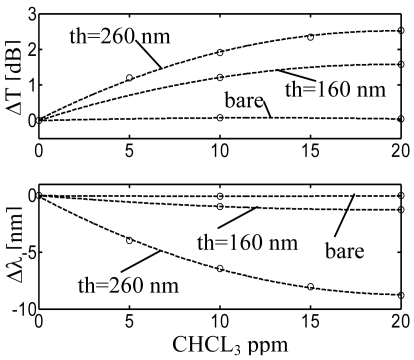
Comparison of the sensitivities, in terms of attenuation band wavelength shift and peak loss reduction, for a bare, a 160 nm coated and a 260 nm coated LPG.

With regard to sensor sensitivity, Figure 24 shows the wavelength shift and amplitude changes versus chloroform concentration with quadratic interpolation for the 160 nm and 260 nm coated LPG compared with the uncoated one. As expected, without the sensing overlay negligible variations have been observed in both the measured parameters. Sensitivities of −0.130 nm/ppm and 0.163 dB/ppm were observed for the thinner overlay, in the range 0–10 ppm. Sensitivities of −0.85 nm/ppm and 0.26 dB/ppm were measured for the thicker overlay, in the same range.

Based on these results, it is evident how the nanoporous polymer layer is the responsible for the significant changes observed in the attenuation band, already in the presence of few *ppm* of pollutants in water. Moreover a sensitivity improvement of more than six times in terms of wavelength shift was obtained in the range 0–10 ppm by considering an overlay with higher thickness and an higher order cladding mode.

A great feature of the proposed configuration is the possibility to select the overlay thickness and mode order in order to meet specific requirements in terms of sensitivity and response time. In conclusion, the phenomenon of the modal transition in HRI thin film coated LPGs and the use of the δ form s-PS as chemo-sensitive material enables sub ppm chemical detection of VOCs with commercially available and low cost spectrometers.

## 6. Chiral Sensors

Several methods for sensing chirality based on racemic host receptors interacting with target non-racemic guests have been proposed. For chromophore racemic receptor molecules, their non-covalent bonding to a non-racemic guest can provide induced circular dichroism (ICD) in the absorption region of the receptor.

Particularly suitable are macromolecular receptors, which are able to form regular helices, and hence in most cases are stereoregular [115-122] In fact, racemic polymers can lead not only to detection but also to amplification of chirality, since cooperative interactions with low-molecular-mass non-racemic compounds can generate prevalence of one polymer helical hand. These chirality transfer and amplification phenomena have been generally observed in solution [115-122].

The occurrence of transfer and amplification of chiral information is particularly relevant for solid polymer films, since they could have in principle applications in chirooptical devices and data storage systems. Recently, is has been reported that non-racemic s-PS films are able to detect, amplify and memorize the chirality of several volatile organic molecules [123,124].

**Figure 25.**
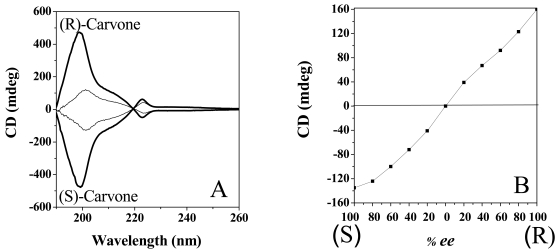
(a) CD spectra of s-PS films, obtained by spin-coating, after exposure to vapor of R or S-carvone. The spin-coating process has been conducted from 1 wt% solutions onto quartz surface at the spin-rate of 1,600 rpm, by using different solvents: chloroform (thick line); tetrahydrofuran (thin line). (b) ICD intensity of the Cotton band at 200 nm for s-PS films spin-coated from a chloroform solution, after exposure to carvone with different enantiomeric excess.

The induced circular dichroism (ICD) of these s-PS sensing films always presents a major Cotton band at 200 nm and a minor Cotton band of opposite sign at 223 nm, but their intensity is critically dependent on the film processing. In particular maximum ICD intensities (in the presence of all the considered non-racemic molecules) have been observed for s-PS films spin-coated from chloroform and tetrahydrofurane solutions, for spin rates larger than 1,600 rpm (Figure 25A). The ICD phenomena are instead negligible for spin-coating procedures with most solvents and always for spin rates lower than 100 rpm [124].

The ICD phenomena, of course, depend also on the enantiomeric excess (*ee*) of the chiral guest. Just as an example, the ICD intensity of the Cotton band at 200 nm for s-PS films spin-coated from a chloroform 0.25 wt% solution at a spin rate of 1,600 rpm, after exposure to carvone is reported in Figure 25B versus the % *ee* of the guest. The change of the ICD intensity is roughly linear with the enantiomeric excess of the temporary guest.

The observed ICD phenomena remain in the s-PS films not only after complete guest removal but also after thermal procedures leading to transitions from the nanoporous δ phase toward the dense helical (γ) and trans-planar (α) crystalline phases. The memory of the volatile non-racemic guest molecules can be erased only by thermal treatments at temperatures higher than the s-PS melting temperature (≈270 °C) or by long-term treatments with strong s-PS solvents [123,124].

These results indicate that the observed ICD phenomena (and hence the chiral memory) are not associated with non-racemic molecules but with the formation of non-racemic supramolecular (possibly crystalline) structures [124]. The obtained s-PS films are suitable not only for detection but also for memory of nonracemic molecules and hence suggest that could be possibly used for chiro-optical memories.

## 7. Conclusions

The first part of this review collects basic information on the polymorphic behavior of s-PS. The emphasis has been mainly given to the crystalline structure of the two nanoporous δ and ε crystalline phases, exhibiting empty space distributed as ordered cavities and channels, respectively. Basic concepts relative to the structure of the corresponding co-crystalline phases, as obtained by sorption of suitable low-molecular-mass guest molecules, have been also given. Moreover, the detailed information relative to the three possible uniplanar orientations that can be achieved for all nanoporous and co-crystalline phases of s-PS has been summarized.

Relevant information relative to the transport properties of vapors and gases into semicrystalline s-PS films has been presented in the Section 3. A relevant sorption capacity of the nanoporous crystalline phases has been observed for many low molecular weight substances. In most cases, the contribution of sorption in the crystalline phase is prevalent and becomes largely dominant in the limit of low activities of the analyte. The reported sorption and desorption kinetics also show that, at low guest activities, the guest diffusivity can be also controlled by a suitable selection of the kind of uniplanar orientation of the host crystalline phase. An additional very relevant feature of the nanoporous crystalline polymeric films is that the analyte uptake (because preferably as guest of the crystalline phase) occurs in the absence of any significant changes of glass transition temperature and elastic modulus, which are generally detrimental to sensing films. Moreover, the sorption mechanism based on the formation of cocrystalline phases between low-molecular-mass molecules and host polymeric crystalline phases involves the molecular selectivity, typical of host-guest compounds.

Application of semicrystalline s-PS films as sensing element of gravimetric QCM sensors has been illustrated in the Section 4, mainly as for the detection of chloroform concentration in the vapor phase or in water solution. The sensitivity of the device has been found to be markedly higher than in the case of a-PS coated QCM. This difference in sensitivity has been attributed to the high sorption capacity of the nanoporous crystalline phase, and markedly increases in the limit of extremely low solvent concentrations in the environment.

Section 5 describes as nanometric thin films of s-PS in the empty nanoporous *δ* form have been successfully integrated with fiber optic technology for the realization of chemical sensors able to detect few parts per million VOCs pollutants both in air and water environments. Two types of fiber optic sensing platforms were exploited both based on the refractive index change of the sensitive s-PS layer upon analyte sorption. The first one makes use of a cleaved fiber optic ended with an s-PS thin film interrogated in reflectometric configuration. The second one exploits Long Period Gratings whose cladding is overlayered by thin films of s-PS. In both cases the use of the δ form s-PS as chemo-sensitive material enables sub ppm chemical detection of VOCs with commercially available and low cost spectrometers.

Unexpectedly, non-racemic s-PS films, if prepared by suitable procedures, are also able to detect, amplify and memorize the chirality of several volatile organic molecules. These films, whose preparation and properties are reviewed in the final section of this review, could have in principle applications not only as sensing elements of non-racemic volatile molecules but also in chirooptical devices.

## Figures and Tables

**Table 1:** Mean and standard deviation for experimental equilibrium response of QCM sensor.

**CHCl_3_ conc. (ppm)**	**Mean Δfreq**	**±σ**	**±σ_%_**
5	−14.9	±0.6	±4.03
10	−26.4	±1.0	±3.79
15	−35.7	±1.4	±3.92
20	−43.3	±1.6	±3.70
25	−49.5	±1.8	±3.64
30	−55.0	±1.9	±3.45
